# Detection of *van*A-Containing *Enterococcus* Species in Faecal Microbiota of Gilthead Seabream (*Sparus aurata*)

**DOI:** 10.1264/jsme2.ME11346

**Published:** 2012-04-28

**Authors:** Joana Barros, Margarida Andrade, Hajer Radhouani, Maria López, Gilberto Igrejas, Patricia Poeta, Carmen Torres

**Affiliations:** 1 Center of Genetics and Biotechnology, University of Tras-os-Montes and Alto Douro, Vila Real, Portugal; 2 Veterinary Science Department, University of Trás-os-Montes and Alto Douro, Vila Real, Portugal; 3 Institut for Biotechnology and Bioengineering, University of Trás-os-Montes and Alto Douro, Vila Real, Portugal; 4 Center of Studies of Animal and Veterinary Sciences, Vila Real, Portugal; 5 Biochemistry and Molecular Biology Area, University of La Rioja, Logroño, Spain

**Keywords:** *Enterococcus*, *vanA*, MLST, fish

## Abstract

Vancomycin-resistant *Enterococcus faecalis*, *E. faecium* and *E. durans* isolates with the genotype *vanA* were detected in 7 of 118 faecal samples (5.9%) of natural gilthead seabream recovered off the coast of Portugal, and one vancomycin-resistant isolate/sample was further characterized. The genes *erm*(B), *tet*(L), *tet*(M), *aac*(6′)-*aph*(2″), *aph*(3′)-IIIa and/or *ant*(6)-Ia were identified in most of the 7 vancomycin-resistant enterococci. Sequence types ST273, ST313 and ST76 were detected in three *E. faecium* isolates and ST6 in two *E. faecalis* isolates. *Van*A-containing enterococci are suggested to be disseminated in fish in marine ecosystems close to areas of human activity.

Enterococci colonize the intestinal tract of humans and many animals and are also commonly found in food, soil and water, representing faecal contamination. Moreover, enterococci have emerged in recent years as important causes of human infections. *Enterococcus* is intrinsically resistant to many antimicrobial agents, but this microorganism can acquire resistance to many other agents. Vancomycin-resistant enterococci (VRE) mediated by the VanA mechanism are an important issue in human health, because they are associated with high-level resistance to vancomycin and teicoplanin, and are usually mediated by conjugative plasmids capable of spreading widely (23). The *van*A cluster of genes is included within the transposon Tn*1546*, which shows heterogeneity in the presence of insertion sequences, deletions and point mutations (12, 25).

*Van*A-containing enterococci have been reported in human clinical samples, but also in non-human sources, such as healthy animals, food and water (15, 17, 19, 24). In a previous study *van*A-containing *E. faecium* isolates belonging the high-risk clonal complex CC17 were also detected in wild mullet fish from the Duero River (1). This type of fish is normally not consumed by humans, inhabits polluted areas and can be used as a sentinel or bioindicator of pollution.

The gilthead seabream (*Sparus aurata*) is a marine teleost fish commonly found in temperate and tropical waters, and represents a key element in the coastal marine ecosystem. Sparids are also of great importance for fisheries and aquaculture. Some studies have demonstrated that their enteric microbiota depend mainly on feeding or the habitat (5). The present study aimed to determine the faecal carriage of VRE in *Sparus aurata* obtained in a natural marine ecosystem close to a urban area, and to carry out the genetic characterization of recovered isolates, analysing the antimicrobial resistance mechanisms implicated, the genetic structures which carry resistant genes and the genetic lineages of isolates as well as the harboured virulence genes.

The presence of VRE was studied in 118 faecal samples of the tested fish. Gilthead-seabream were captured in September–November 2007 in the Atlantic Ocean (specifically, off the Peniche coast, a city in Portugal with 15,600 inhabitants) by sport fishermen using a hand line technique. Small and large intestines of each animal were collected and they were opened with sterile scissors and transferred to sterile Stomacher bags with 5 mL sterile saline solution. A sterile swab was placed in the solution, after agitation, and spread onto a Slanetz-Bartley agar plate (Scharlau) supplemented with 4 mg L^−1^ vancomycin, and then incubated for 48 h at 35°C. Colonies with typical enterococcal morphology were identified to the genus and species level by their cultural characteristics, Gram stain, catalase test, bile-aesculin reaction and biochemical tests using the system API ID20 Strep (BioMérieux, La Balme Les Grottes, France). Species identification was confirmed by species-specific PCR using primers and conditions previously reported (21). Susceptibility to 11 antimicrobials was tested (vancomycin, teicoplanin, ampicillin, streptomycin, gentamicin, kanamycin, chloramphenicol, tetracycline, erythromycin, quinupristin-dalfopristin and ciprofloxacin) by the agar disc diffusion method (3). The minimal inhibitory concentration (MIC) to vancomycin and teicoplanin was determined by the agar dilution method as previously recommended (3). *Enterococcus faecalis* ATCC 29212 and *Staphylococcus aureus* ATCC29213 were used as quality control strains.

Vancomycin resistance genes (*van*A, *van*B, *van*C-1, *van*C-2/3 and *van*D) were sought by PCR in enterococcal isolates which showed resistance or reduced susceptibility to glycopeptides (21), and positive amplicons were sequenced. Resistance genes for other antimicrobials, including macrolides [*erm*(A), *erm*(B), *erm*(C)], tetracycline [*tet*(M), *tet*(K), *tet*(L), *tet*(S), *tet*(O)], aminoglycosides [*aph*(3′)-IIIa, *aac*(6′)- *aph*(2″), *ant*(6)-Ia], streptogamines [*vat*(D) and *vat*(E)], and chloramphenicol [*cat*(A)], were also tested by PCR in all the enterococcal isolates that showed resistance or reduced susceptibility for these agents, using primers and conditions previously reported (10, 21). Positive and negative control strains from the collection of the University of La Rioja (Spain) were used in all PCRs. The presence of the virulence genes *esp* and *hyl* was detected by PCR, using previously described primers and conditions (13).

VRE with an acquired mechanism of resistance were recovered from 7 of the 118 faecal samples (5.9%) collected from gilthead seabream and one vancomycin-resistant isolate per positive sample was further studied (7 VRE isolates from the 7 positive samples). The *van*A gene was detected in all 7 VRE, and all showed the typical VanA phenotype, including high-level resistance to both vancomycin and teicoplanin (MIC≥128 and 16–64 mg L^−1^, respectively). The enterococcal species found were as follows: *E. faecium* (3 isolates), *E. faecalis* (2 isolates) and *E. durans* (2 isolates) (Table 1).

VRE isolates obtained showed resistance, in addition to glycopeptides, also to ampicillin, ciprofloxacin, erythromycin, high-level streptomycin, gentamicin, or kanamycin, tetracycline, quinupristin-dalfopristin and chloramphenicol, with different phenotypes of resistance (Table 1). Furthermore, four VRE showed a multi-resistance phenotype (including three or more families of agents), highlighting one *E. faecalis* that showed resistance to seven different groups of antimicrobial agents. The *erm*(B) gene was found in all 5 erythromycin-resistant *van*A isolates of this study. The *tet*(L) or *tet*(M) genes (separate or combined) were identified in two of the four tetracycline-resistant *van*A-containing isolates: *tet*(M)+*tet*(L) (1 *E. durans*); and *tet*(L) (1 *E. faecalis*) (Table 1). These genes were detected in previous studies in tetracycline-resistant *van*A isolates from animals (24). The genes *tet*(M), *tet*(K), *tet*(L), *tet*(S), and *tet*(O) were negative in the remaining two tetracycline-resistant VRE isolates (C2520 and C2525); we cannot reject the possible presence in these isolates of other known *tet* genes or even novel *tet* genes. Future studies will clarify this point. The *aph*(3′)-IIIa, *aac*(6′)-*aph*(2″) and *ant*(6)-Ia genes, which codify resistance to high-level kanamycin, gentamicin and streptomycin agents, respectively, were detected in two of our *E. faecalis* isolates.

The wide dissemination of antimicrobial resistance in enterococci has been attributed to both clonal dissemination of isolates and horizontal transfer of mobile genetic elements containing vancomycin-resistant genes. It is well known that the *vanA* cluster, which carries nine genes mediating resistance to both vancomycin and teicoplanin, is contained as part of the Tn*1546*-like elements (25). Characterization of Tn*1546* element in our 7 *vanA*-positive enterococci was carried out by overlapping PCRs with primers and conditions previously reported (2, 12, 16), based on the Tn*1546* prototype (GenBank M97297), and named structure type I in this work. Amplicons obtained that showed longer or shorter sizes than expected according to type I were sequenced to determine the type and position of the possible insertion sequences or deletions (12). All our *van*A-containing isolates presented the complete structure of transposon Tn*1546*, without insertions or deletions, identical to the prototype (GenBank M97297), except for *van*A-containing *E. faecium* C2519 strain, which presented the insertion sequence IS*Ef1* in the intergenic region *van*X-*van*Y at position nt9044. The presence of this insertion sequence at the same position in Tn*1546* has been previously reported in *vanA*-containing enterococci recovered from different origins, mostly clinical samples, in Portugal and Spain (12, 16).

The Multi-Locus-Sequence-Typing (MLST) scheme was used to investigate the epidemiology and population structure of the 5 *vanA*-containing *E. faecalis* and *E. faecium* isolates detected in this study. For this purpose, internal 400–600 bp fragments of 7 housekeeping genes (*adk*, *atp*A, *ddl*, *gdh*, *gyd*, *pur*K, and *pst*S for *E. faecium*; and *gdh*, *gyd*, *pst*S, *gki*, *aro*E, *xpt* and *yiq*L for *E. faecalis*) were amplified and sequenced (8, 20). The sequences obtained were analysed and compared with the database (http://www.mlst.net). A specific sequence type (ST) and clonal complex (CC) were assigned after analyzing the combination of the seven obtained alleles.

The sequence types detected among the 3 *van*A-containing *E. faecium* isolates were as follows: ST76 (*E. faecium* C2521), ST273 (*E. faecium* C2519) and ST313 (*E. faecium* C2520). The sequence types ST273 and ST313 are single *locus* variants of ST18 and are included in the high-risk clonal complex (HRCC) CC17. This HRCC has been associated with *E. faecium* isolates that are well adapted to the hospital environment, and frequently associated with ampicillin and ciprofloxacin resistances, and with the presence of the *esp* virulence gene (7, 9). It is interesting to note that our two *E. faecium* CC17 strains were *esp*-negative and showed susceptibility to ciprofloxacin, and only one showed ampicillin resistance (Table 1). This CC17 has been also detected in environmental strains in other studies performed with isolates of buzzard faeces samples (18), and also in *van*A-containing *E. faecium* isolates obtained from mullet fish from the Duero River in a previous study (1). The sequence type ST76 belongs to a small complex of 4 STs (ST75, ST76, ST77 and ST218).

The two *van*A-containing *E. faecalis* strains in this study (C2525 and C2527) were ascribed to sequence type ST6, included in CC2. This clonal complex is particularly associated with the hospital environment (6, 11) and has been also defined as an HRCC (20). The origin of *E. faecium* and *E. faecalis* strains of HRCC in the intestinal tract of marine fish was not clear of this study and we cannot reject contamination of the marine coastal area with human effluent.

The virulence genes *esp* or *hyl* were checked by PCR (13) in our VRE strains, and all gave negative results (Table 1). There are very few reports about the presence of virulence factors in enterococcal isolates of food and/or animal faecal samples (4, 13), and these genes have been specially reported in clinical isolates.

In conclusion, marine fish can carry *van*A-containing enterococci of diverse species and it is interesting that *E. faecium* and *E. faecalis* strains ascribed to HRCC, (CC17 and CC2, respectively), usually associated with the hospital setting (11, 14, 22), were detected among them. The *van*A resistance gene seems to be widespread in enterococci of natural ecosystems, in addition to clinical settings, and it would be interesting to track the routes of dissemination of these resistant microorganisms that have great importance in public health. The information obtained in this study is important to understand the evolution of antimicrobial resistance in aquatic environments and the potential link of different ecological niches in relation to this issue.

## Figures and Tables

**Table 1:** Characteristics of vancomycin-resistant enterococcal strains recovered from *Sparus aurata* in Portugal

Strain	MIC (mg L^−1^)^a^		Vancomycin resistance mechanism	Sequence type (clonal complex)	Antimicrobial resistance phenotype^a^	Other resistance genes^c^

	VAN	TEI				
*E. faecium* C2519	>128	64	*van*A	ST273 (CC17)	ERY-AMP-CIP	*erm*(B)
*E. faecium* C2520	>128	64	*van*A	ST313 (CC17)	ERY-TET^b^	*erm*(B)
*E. faecium* C2521	>128	64	*van*A	ST76 (singleton)	ERY	*erm*(B)
*E. durans* C2523	>128	64	*van*A		QD	—
*E. durans* C2524	>128	64	*van*A		TET-CHL-QD	*tet*(M)+*tet*(L)
*E. faecalis* C2525	>128	64	*van*A	ST6 (CC2)	ERY-TET^b^-CHL-QD-CIP-STR-GEN-KAN	*erm*(B)+*ant*(6)-Ia+*aac*(6′)-*aph*(2″)+*aph*(3′)-IIIa
*E. faecalis* C2527	>128	64	*van*A	ST6 (CC2)	ERY-TET-QD-CIP-KAN	*erm*(B)+*tet*(L)+*aph*(3′)-IIIa

a VAN, vancomycin; TEI, teicoplanin; AMP, ampicillin; STR, high-level streptomycin; KAN, high-level kanamycin; GEN, high-level gentamicin; TET, tetracycline; ERY, erythromycin; CIP, ciprofloxacin; QD, quinupristin–dalfopristin; CHL, chloramphenicol.

b The genes *tet*(M), *tet*(K), *tet*(L), *tet*(S), and *tet*(O) were negative in this strain.

c The virulence genes *esp* or *hyl* were negative in all strains
